# Gene expression profile of peripheral blood mononuclear cells in response to HIV-VLPs stimulation

**DOI:** 10.1186/1471-2105-9-S2-S5

**Published:** 2008-03-26

**Authors:** Luigi Buonaguro, Alessandro Monaco, Eleonora Aricò, Ena Wang, Maria Lina Tornesello, George K Lewis, Franco M Marincola, Franco M Buonaguro

**Affiliations:** 1 Lab. Viral Oncogenesis and Immunotherapies & AIDS Reference Center, Department of Experimental Oncology, Istituto Nazionale Tumori “Fond. G. Pascale”, 80131 Napoli, Italy; 2 Immunogenetics Section, Department of Transfusion Medicine, Clinical Center, National Institutes of Health, Bethesda, MD 20892-1502, USA; 3 Department of Cell Biolology and Neurosciences, Istituto Superiore di Sanità, Rome, Italy; 4 Institute of Human Virology, University of Maryland School of Medicine, University of Marlyand, Baltimore, MD 21201, USA

## Abstract

**Background:**

Baculovirus-expressed HIV-1 Pr55gag Virus-Like Particles (HIV-VLPs) induce maturation and activation of monocyte-derived dendritic cells (MDDCs) with a production of Th1- and Th2-specific cytokines.

**Results:**

The analysis of genomic transcriptional profile of MDDCs, obtained from normal healthy donors and activated by HIV-VLPs, show the modulation of genes involved in the morphological and functional changes characterizing the MDDCs activation and maturation. Similar data are obtained using peripheral blood mononuclear cells (PBMCs), without further selection, showing the feasibility of a direct and “simplified” experimental procedure.

**Conclusions:**

The results here described show that the maturation pattern induced by HIV-VLPs in *ex vivo* generated MDDCs, can be observed also in CD14-expressing freshly derived PBMCs, with the possible identification of genetic predictors of individual response to immunogens.

## Introduction

Dendritic cells (DCs) are professional antigen-presenting cells (APC) able to initiate immune responses [1,2]. Immature DCs are located in peripheral tissues to continuously monitor the environment through the uptake of particulate and soluble products. Antigen-loaded DCs acquire a mature phenotype, associated with reduced endocytic and phagocytic capacities [3-6], and migrate toward the lymphoid organs to activate naïve T cells, through upregulated costimulatory molecules such as CD40, CD80, CD83 and CD86 [7]. This effect is elicited by the recognition and binding of pathogen-associated molecular patterns (PAMPs) to pathogen-recognition receptors (PRRs) expressed on the DCs, including Toll-like Receptors (TLRs) and C-type lectins [8-10].

There are two main DC types in human peripheral blood, known as myeloid DCs (mDCs), the major subset representing around 80% of blood DCs [11], and plasmacytoid DCs (pDCs). However, considering that DCs represent only 1-3% of peripheral blood mononuclear cells (PBMCs), immature DCs can be obtained *in vitro* from peripheral blood monocytes, generating monocyte-derived DCs (MDDCs) [12]. Additional professional APCs in PBMCs are represented by Macrophages and B-cells.

The analysis of the transcription profile, defined as transcriptome, may be highly informative of the molecular basis underlying the morphological, phenotypical and functional changes of APCs induced by immunogens. In particular, the expression pattern of specific sets of genes upon DC differentiation and maturation has been reported, showing a great plasticity of the DC transcriptional programs, activated in response to CD40L, LPS and cocktail of inflammatory cytokines and prostaglandin (PG) E(2) (CyC) [13,14]. Furthermore, a time-specific kinetic of response has been observed in MDDC activated with pathogen components, showing a rapid upregulation of genes associated with the innate arm of the immune response, followed by induction of adaptive immune response genes [15-17].

Virus-like particles (VLPs) represent a peculiar form of subunit vaccine based on viral capsid and envelope proteins which show the ability to self-assemble into highly organized particulate structures resembling immature virus particles [18,19]. VLPs can deliver antigenic structures, such as whole proteins or specific individual epitopes and have been shown to generally induce more effective humoral and cellular immune response than their soluble counterparts [20].

The VLPs developed in our laboratory are based on the Human Immunodeficiency Virus type 1 Pr55gag precursor protein (HIV-VLPs) and present an entire gp120 molecule from a Subtype A HIV-1 Ugandan isolate, anchored through the trans-membrane (TM) portion of the Epstein-Barr virus (EBV) gp220/350 [21-23].

The HIV-VLPs show a strong in vivo immunogenicity in Balb/c mice, even in absence of adjuvants, and HIV-1-specific T cell response (CD4+ and CD8+) as well as cross-clade neutralizing antibodies have been detected in immunized animals, at systemic as well as local (vaginal and intestinal) level [24,25]. These properties suggest the ability to promote the activation of antigen-presenting cells (APCs) and a cross-presentation of peptides in association to both MHC class I and -II molecules [26,27].

We have recently shown that baculovirus-expressed HIV-VLPs are able to induce maturation of MDDCs, resulting in expression of surface maturation markers as well as increased production of Th1 polarizing cytokines [28]. Moreover, the HIV-VLP-activated MDDCs show specific changes in the transcriptional profile of genes involved in the morphological and functional changes characterizing the MDDCs activation and maturation [29].

Here we show changes in the gene expression of PBMCs activated with the baculovirus-expressed HIV-VLPs developed in our laboratory, in order to compare their transcriptional profiles with the one observed in *ex vivo* generated MDDCs. A validation of this approach would greatly facilitate the screening of immunogenetic analyses performed on subjects to be enrolled in vaccination programs.

## Materials and methods

### Cell culture medium

DC culture medium consisted of RPMI 1640 medium (Life Technologies, Carlsbad, Calif.) supplemented with 2 mM L-glutamine (Sigma), 1% nonessential amino acids (Life Technologies), 1% sodium pyruvate (Life Technologies), 50 μM 2-mercaptoethanol (Sigma), 50 μg of gentamicin (Life Technologies) per ml, and 10% fetal calf serum (Life Technologies).

### DC preparation and treatment

Monocyte-derived DCs were generated as described previously [6], with minor modifications. Briefly, human peripheral blood mononuclear cells were isolated, from three independent normal healthy donors, by Ficoll-Hypaque density gradient centrifugation and were enriched for CD14^+^ monocytes by negative selection with a cocktail of monoclonal antibodies from StemCell Technologies (Vancouver, British Columbia, Canada), according to the instructions of the manufacturer. Typically, greater than 80% of the cells were CD14^+^ after enrichment (data not shown). The isolated monocytes were allowed to adhere to plastic by plating 10^6^ cells per/ml in RPMI 1640 medium for 2 h. Adherent monocytes were washed with RPMI 1640 medium and were then cultured for 6 days at 10^6^ cells per/ml in DC culture medium supplemented with 50 ng of recombinant GM-CSF (rGM-CSF, R&D Systems, Minneapolis, Minn.) per ml and 1,000 U of recombinant IL-4 (rIL-4; R&D Systems, Minneapolis, Minn.) per ml.

After 6 days in culture, MDDCs were pulsed with 5μg/ml of HIV-VLPs for 8 hours, for gene microarray analysis, and for 16 hours for maturation and activation phenotype analysis. In parallel, PBMCs isolated by Ficoll-Hypaque density gradient centrifugation from same normal healthy donors, were pulsed with same concentration of HIV-VLPs for 12 hours.

### Analysis of DC phenotype

MDDCs and PBMCs were incubated for 30 min at 4°C with murine monoclonal antibodies specific for CD80, CD83, CD86, HLA-DR and CD14 [PBMCs] (BD Pharmingen, San Diego, CA), washed and then fixed with 2% paraformaldehyde for analysis with a FACScalibur flow cytometer (BD Pharmingen). Data analysis was carried out with FlowJo software (Tree Star Inc., San Carlos, CA). The cell fraction that responded by upregulation of activation markers on the cell surface was calculated by overlaying the histograms of treated and untreated cells and Overton subtraction of the curves.

### RNA preparation and microarray hybridization

DCs were harvested, washed twice in PBS and lysed in 350ul RLT buffer with fresh addition of 2-Mercaptoethanol per each well of the 6-well plate. Total RNA was isolated using RNeasy minikits (Qiagen), according to the manufacturer's protocol, and RNA quality and quantity was estimated by Agilent Bioanlayzer (Agilent Technologies, Palo Alto, CA) and NonoDrop. Amplified antisense RNA (aRNA) was obtained from total RNA (0.5-3 µg) via two round of in vitro transcription, according the protocol previously described [30]. 6ug of amplified test samples aRNA were labeled with Cy5 (Amersham) while the same amount of reference sample (pooled normal donor PBMCs) was labeled with Cy3. Test-reference sample pairs were mixed and co-hybridized to 17K cDNA microarrays [31].

### Microarrays and statistical analyses

Hybridized arrays were scanned at 10-µm resolution on a GenePix 4000 scanner (Axon Instruments) at variable PMT voltage to obtain maximal signal intensities with less than 1% probe saturation. Resulting jpeg and data files were deposited at microarray data base (mAdb) () and retrieved after median centered, filtering of intensity (>300) and spot elimination (bad and no signal). Data were further analyzed using Cluster and TreeView software [31] and Partek Pro software (Partek). Subsequent low-stringency filtering (80% gene presence across all experiments and at least one experiment with ratio fold change >3), 3,119 genes were selected for further analysis. Hierarchical cluster analysis was conducted on these genes according to Eisen *et al*. [32]; differential expressed genes were visualized by Treeview and displayed according to the central method [33].

### Ethical issues

All human specimens were obtained under informed consent, as approved by the University of Maryland Baltimore Institutional Review Board.

## Results

### Baculovirus-HIV-VLP induces a maturation phenotype of APCs

**Figure 1:**
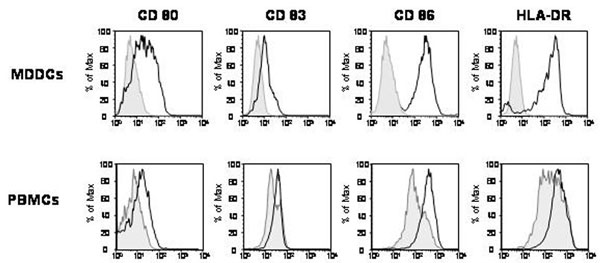
**Maturation of DCs by baculovirus-expressed HIV-VLPs**. Immature MDDCs and PBMCs were incubated in the presence of the HIV-VLPs for 16 and 12 hours, respectively. The expression of CD80, CD83, CD86 and HLA-DR was analyzed on fixed cells by FACScalibur flow cytometer and data analysis was carried out with FlowJo software. The PBMCs were gated for the CD14 positivity. The results of a representative experiment are shown; the shadowed curve represents the untreated cells.

Immature MDDCs and freshly derived PBMCs were obtained from the same three independent donors and were incubated with 5 μg/ml of HIV-VLPs. After a 8-12 hr-induction, the expression of surface maturation/activation markers, such as CD80, CD83, CD86 and HLA-DR, was examined. The expression of all the four markers was upregulated in MDDCs as well as in CD14 – expressing PBMCs by treatment with HIV-VLPs, compared to PBS (Fig.1). In particular, CD14+ cells are identified as common, immediate myeloid DC precursors with the ability to differentiate into interstitial DC (IDC) and Langerhans cells (LC) [34].

### Pattern of MDDCs and PBMCs response to HIV-VLPs

Gene expression profiles were generated from HIV-VLP-treated MDDC and PBMCs (defined together from now on, as DCs). Amplified antisense RNA (aRNA) was obtained from total RNA extracts [30] and hybridized to a custom-made 17,000 (17K)-clone cDNA microarray chip enriched with genes relevant to immune function. Stringent filtering were further applied to eliminate genes with missing value in >20% of all the experiments and >3 fold change in at least one experiment. The remaining 3,119 genes were thus used for statistic analysis.

**Figure 2:**
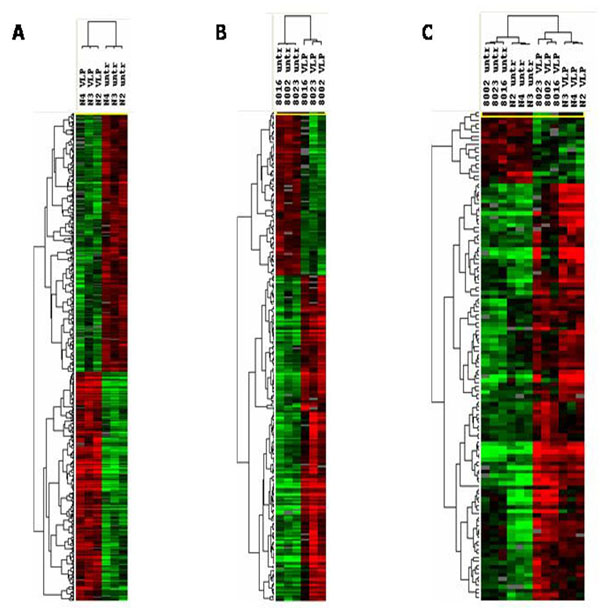
**Supervised hierarchical clustering of genes differentially expressed in HIV-VLP-treated cells**. The clusterograms represent an Eisen hierarchical clustering of genes differentially expressed (p< 0.005) in HIV-VLPs-treated PBMCs (A), MDDCs (B). The comparison between the two cell populations is shown in C. The clustering is defined by the dendrogram on the top of the clusterogram.

Supervised cluster analysis obtained on either MDDCs or PBMCs samples stimulated with HIV-VLPs shows the segregation of the untreated from the HIV-VLP-treated samples, at a significance threshold of univariate test <0.005 (Fig. 2A and B). The same analysis, performed using all the data obtained on both cell populations, show two distinct clusters with a clear segregation of untreated samples from the HIV-VLP-treated MDDCs and PBMCs (Fig. 2C). This result indicates the identification of a similar transcription machinery induced in both *ex vivo* purified MDDCs and *in vivo* “unselected” PBMCs.

### Gene expression changes induced in DCs by HIV-VLP treatment

The differential gene expression in HIV-VLP-treated DCs, compared to untreated samples, was considered statistically significant only when supported by a p<0.005, and treatment–induced changes in gene profiling were analyzed using Student's t test. Considering only genes showing at least a 1.5-fold modulation (increase or reduction) in the transcriptional levels, it has been possible to identify unique genes in the profile induced by HIV-VLPs (Table 1).

**Table 1:** Pathways involved in the HIV-VLPs-induced PBMCs. The pathways are derived from the BioCarta through the Cancer Genome Anatomy Project at . Genes with at least a 1.5-fold modulation (up or downregulation) have been taken into consideration.

**Pathways**	**VLP vs PBS Up-regulated**		**Pathways**	**VLP vs PBS Down-regulated**
**Antigen processing and presentation**	CTSL		**ECM-receptor interaction**	CD36
**Cell cycle, proliferation, cell death**	IL3RA SMOX BCL2 G0S2 IER3		**Cytokine-cytokine receptor interaction**	IL1R2
**Cell shape & extracellular matrix**	SERPINB2 LIMK2		**Chaperones modulate interferon Signaling Pathway**	HSPA7
**Chemokine and cytokines**	IL6 PBFE1 PBX3			
**Cytokine Network**	IL1-A IL1-B			
**Cytokine-cytokine receptor interaction**	CCL18 CCL20 CCL7 CXCL1 CXCL2 CXCL3 CXCL6 CXCL13 INHBA			
**Immune response**	ACTN1			
**Membrane proteins**	AQP9 EMR1 SLC25A37 SLCO4A1			
**Transcription**	MAD			
**Selective expression of chemokine receptors during T-cell polarization/Toll-like receptor signaling pathway**	CCL4 (MIP1β) CCL3 (MIP 1α) IL8			

The HIV-VLP treatment induced in PBMCs the upregulation of 58 genes and the downregulation of 7 genes, indicating that a specific reprogramming of the transcriptional profile is observed in PBMCs, presumably in the Antigen-Presenting Cell (APCs) populations. This observation confirms our previous report, showing that HIV-VLPs induce a specific transcriptional profile pattern in MDDCs, distinct from either PBS or LPS treatment [29].

**Figure 3:**
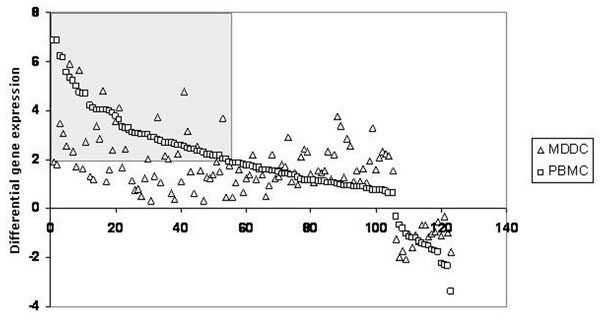
**Genes with a differential expression identified in both HIV-VLP- treated MDDCs and PBMCs**. The level of differential expression for each of the common genes identified in both analyses is shown. The gray boxes include the genes showing a differential expression >2 in both MDDCs and PBMCs induced with HIV-VLPs.

In particular, merging the genes modulated in the analyses performed on MDDCs and PBMCs, a significant set (>100) of common genes with a differential (up or down regulation) transcription has been identified. Moreover, a specific subset of these genes (>25) shows a differential transcription >2 in both analyses, suggesting the recognition of a strong transcriptional “signature” pattern common to both cell target populations (Fig. 3).

### Pathways modulation in PBMCs in response to HIV-VLPs

The PBMCs transcriptional profile was analyzed on the basis of the cellular pathways modulated by the HIV-VLP-treatment. In respect to the main focus of this study, only the pathways involved in immune activation have been evaluated in more detail.

The HIV-VLPs induction of PBMCs results in the activation of genes associated with antigen presentation functions. A set of cytoskeletal genes that may potentially mediate shape change and migratory behavior of activated APCs is also observed. The increase in the expression of immune cytokines, chemokines, and receptors contribute to the recruitment of monocytes, DCs, and macrophages to the site of infection. Moreover, they modulate both innate and adaptive immune response, including the polarization of Th cells, and the down-regulation of the inflammatory response, which may significantly interfere with the immune response. The induction of signaling genes and transcription factors may be involved in preparing the DC to be receptive to regulatory signals in the lymphatics and lymph nodes. All these categories of genes are extremely similar to what observed by us upon HIV-VLP induction of *ex vivo* derived MDDCs [29].

It is extremely interesting that among the common upregulated genes, identified in the analyses performed on MDDCs and PBMCs, some of them are intimately related to the immune activation (CCL3/MIP-1α, CCL4/MIP-1β, CCL20/MIP-3α, IL1a, IL8). In particular, CCL3, CCL4 and CCL20 are chemokines actively participating in the host response to invading pathogens by regulating the trafficking and activation stage of inflammatory cells, including the Toll-like receptor signaling pathway. They all exert similar effects on monocytes and are potent chemoattractants for lymphocytes and dendritic cells. More specifically, CCL3 selectively attracting CD8+ Tcells, CCL4 selectively attracting CD4+ Tcells and CCL20 promoting the adhesion of memory CD4+ Tcells [35-39].

Collectively, the transcriptional profile induced by HIV-VLPs in freshly drawn PBMCs reflects a significant cellular and immunological reprogramming of cells directly involved in the host immune response.

## Conclusions

The results here described show that the maturation pattern induced by HIV-VLPs in *ex vivo* generated MDDCs, can be observed also in CD14-expressing freshly derived PBMCs. Moreover, the genomic transcriptional profile induced by HIV-VLPs in PBMCs shows the activation of unique genes and cellular pathways, reflecting a distinctive cellular and immunological reprogramming of circulating cells deputed to trigger the host immune response.

A supervised Eisen's clustering analysis confirms the specificity of the observation, given that the PBMCs samples, derived from the HIV-VLP treatment, cluster together indicating that the pattern of specifically modulated genes is consistent all across the analyzed samples. A comparison with the pattern induced by HIV-VLP treatment in *ex vivo* generated MDDCs identifies a cluster of genes whose expression is similarly modulated, indicating that a specific transcriptional signature is found between *ex vivo* generated MDDCs and “unselected” PBMCs.

Among the pathways and specific genes activated in PBMCs treated with HIV-VLPs, those directly involved in the biological functions as antigen presenting cells (APCs) have been analyzed in detail. The functional maturation and activation of CD14-positive cell population present in PBMCs by HIV-VLPs has been shown and, in particular, the activation of genes involved in cellular control (proliferation, differentiation, migration and homeostasis) as well as in functional activity (antigen presentation, T cell activation and Th polarization) has been observed.

These results are extremely interesting, indicating the sensibility and the specificity of the gene transcriptional profile analysis for identifying in PBMCs, regardless the heterogeneity of cell populations unrelated to the host immune response, a cluster of genes whose transcription is specifically modulated in response to an antigen, such as the HIV-VLPs. The cell populations likely involved in this process are the blood DCs (myeloid DCs, mDC; and plasmacytoid DCs, pDCs), as well as the additional professional APCs such as Macrophages and B-cells. Their specific individual involvement in the observed reponse will be further evaluated.

These results confirm the data obtained by HIV-VLP treatment of selected *ex vivo* generated MDDCs [29]. In this regard, the presence of transcriptionally modulated genes common to both analyses strongly suggest the possibility of identifiyng a genomic signature in PBMCs induced by an antigen, without the need of purification and *ex vivo* selection of DCs. If the reported data will be confirmed on a larger scale, the possibility of screening the donor susceptibility to an antigen treatment using PBMCs would greatly simplify the identification of “responsive” vaccinees and the understanding of eventual failures in individuals enrolled in clinical trials.

Microarray approach allows quantitative and simultaneous analysis of gene expression of a large amount of genes and the systematic studies of expression patterns are extremely useful for identify molecular events and key pathways involved in cellular functions induced by specific stimuli. In particular, data on the global pattern of gene expression underlying the modifications induced by HIV-VLPs in PBMCs, at early stages of antigen administration, may be extremely helpful for the identification of exclusive activation markers to trace the biological effects of modifications/optimizations of the HIV-VLP vaccination strategy.

## Competing interests

The authors declare that they have no competing interests.

## Authors' contributions

**LB** carried out experiments on DCs and PBMCs; wrote the article;** AM** carried out gene expression experiments on PBMCs;** EA** carried out gene expression experiments on DCs; **EW** supervised gene expression experiments on PBMCs; **MLT** participated to production of VLPs; **GKL** participated in designing experiments on DCs and PBMCs; **FMM** participated in designing gene expression experiments; **FMB** supervised the overall project.
